# Sphingosine-1-phosphate and its receptors in vascular endothelial and lymphatic barrier function

**DOI:** 10.1016/j.jbc.2023.104775

**Published:** 2023-05-02

**Authors:** Cynthia Weigel, Jacqueline Bellaci, Sarah Spiegel

**Affiliations:** Department of Biochemistry and Molecular Biology Virginia Commonwealth University School of Medicine, Richmond, Virginia, USA

**Keywords:** lymph endothelial cells, S1P receptors, lymphatic barrier functions, S1P, endothelial junctions

## Abstract

The vascular and lymphatic systems both comprise a series of structurally distinct vessels lined with an inner layer of endothelial cells that function to provide a semipermeable barrier to blood and lymph. Regulation of the endothelial barrier is critical for maintaining vascular and lymphatic barrier homeostasis. One of the regulators of endothelial barrier function and integrity is sphingosine-1-phosphate (S1P), a bioactive sphingolipid metabolite secreted into the blood by erythrocytes, platelets, and endothelial cells and into the lymph by lymph endothelial cells. Binding of S1P to its G protein–coupled receptors, known as S1PR1-5, regulates its pleiotropic functions. This review outlines the structural and functional differences between vascular and lymphatic endothelium and describes current understanding of the importance of S1P/S1PR signaling in regulation of barrier functions. Most studies thus far have been primarily focused on the role of the S1P/S1PR1 axis in vasculature and have been summarized in several excellent reviews, and thus, we will only discuss new perspectives on the molecular mechanisms of action of S1P and its receptors. Much less is known about the responses of the lymphatic endothelium to S1P and the functions of S1PRs in lymph endothelial cells, and this is the major focus of this review. We also discuss current knowledge related to signaling pathways and factors regulated by the S1P/S1PR axis that control lymphatic endothelial cell junctional integrity. Gaps and limitations in current knowledge are highlighted together with the need to further understand the role of S1P receptors in the lymphatic system.

## Overview of the vascular and lymphatic systems

The vascular system is a complex network of vessels that transport blood throughout the body to provide tissues with oxygen and nutrients, while also carrying away carbon dioxide and other waste products for excretion. Proper functioning of the circulatory system is critical, and the occlusion of even small vessels can lead to several life-threatening illnesses, including pulmonary embolism, heart attack, and stroke. The vasculature is also involved in a variety of other important biological processes, such as maintenance of blood pressure and body temperature and transport of signaling molecules, nutrients, metabolites, erythrocytes, and immune cells (1).

**Figure 1:**
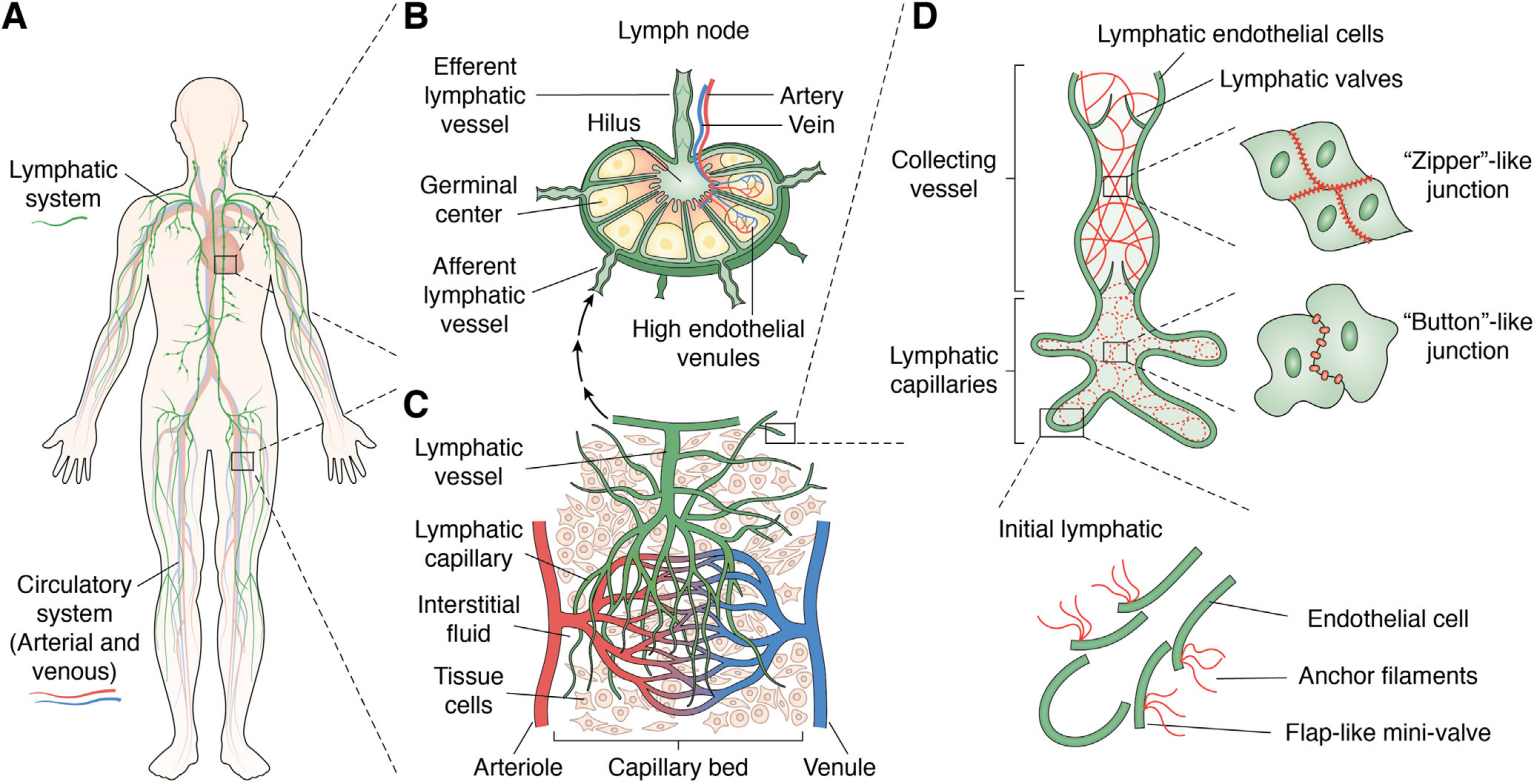
**Schematic representation of the lymphatic system.***A*, overview of the lymphatic system with connections to the circulatory system (arterial and venous) within the body. The lymphatic vessels (*green*) parallel the circulatory system and function to return excess interstitial fluid and proteins to the bloodstream. The lymphatic vessels are also important for immune surveillance and transportation of immune cells. *B*, lymph nodes with efferent lymphatic vessels (*green*), the hilus at which arteries carrying nutrients and lymphocytes enter the lymph node and veins leave it, germinal center, high endothelial venules, and afferent lymphatic vessel. The germinal center is the site of active B-cell proliferation and differentiation. High endothelial venules are specialized blood vessels that allow lymphocytes to enter the lymph node from the bloodstream. Efferent lymphatic vessels leave the lymph node *via* the hilum and carry lymph away from the lymph node, while afferent lymphatic vessels bring the lymph fluid into the lymph node from distant organs. *C*, capillary bed with lymphatic vessel (*green*), lymphatic capillaries, interstitial fluid, tissue cells, arterioles (*red*), and venules (*blue*). The capillary bed is the site of exchange between the bloodstream and the interstitial fluid surrounding tissue cells. Excess interstitial fluid and proteins enter the lymphatic capillary and are transported by the lymphatic vessels. *D*, lymphatic capillary of collecting vessel with lymphatic valves, zipper-like junction between the lining endothelial cells and button-like junction, and initial lymphatic with button-like junction between the endothelial cells. Initial lymphatics are blind-ended vessels and are connected by anchoring filaments to the surrounding. Overlapping endothelial cells build up flap-like mini-valves ensuring a one-way valve system that allows interstitial fluid and proteins to enter but prevents their backflow.

The vessels of the vasculature comprise three histologically distinct layers, or “tunics,” that vary in thickness and composition depending on the vessels’ location and function (1). The outermost layer, the tunica adventitia or externa, is made of connective tissue that secures the vessels to their surroundings (2), and also includes the *vasa vasorum* (intrinsic vessels) and the *nevi vasorum* (nerves) that supply the cells of the vessel itself (2). The tunica media, the middle layer, is made of smooth muscle and extracellular matrix with elastic fibers, proteoglycans, and collagen that can assist in vasoconstriction and vasodilation (3). The tunica intima, the innermost and thinnest layer, consists of a monolayer of endothelial cells (ECs) lining the entire vasculature attached to the basement membrane and the subendothelial extracellular matrix (4). The vessels of the vascular system include arteries, veins, and capillaries that have unique structural features based on their location and function within the body (Fig. 1*A*).

The lymphatic system includes the primary lymphoid organs (bone marrow, thymus), the secondary lymphoid organs (spleen, lymph nodes), and the lymphatic vessels that connect all lymphoid organs (Fig. 1, *A* and *B*). The roles of the lymphatic system are distinct from those of the vascular system, and thus the lymphatic vessels, referred to as lymphatics, display a number of unique structural characteristics that distinguish them from blood vessels (5). The three main functions of the lymphatics are the return of interstitial fluid from the tissue to the circulation, transport of digested lipids, and circulation of immune cells (6). The lymphatics are typically organized into three distinct groups: the initial lymphatics, responsible for the absorption of interstitial fluid; the precollecting vessels; and collecting vessels, which are responsible for the propulsion of lymph fluid (Fig. 1, *C* and *D*).

The initial lymphatics are blind-ended vessels surrounded by layers of smooth muscle cells that assist in the propulsion of lymph and contain bicuspid valves distributed in an irregular pattern (5). The collecting lymphatics possess secondary valves similar to the valves of veins that prevent retrograde flow of lymph toward the initial lymphatics (5) (Fig. 1*D*). From the collecting lymphatics, most of the lymph returns to the circulation through the thoracic duct.

Disorders of the lymphatic systems can lead to a variety of diseases. Damage to the lymph nodes or lymphatics can cause lymphedema, a swelling of the peripheral tissue due to impaired lymphatic drainage and the accumulation of protein- and lipid-rich interstitial fluid (7). Other pathologies associated with the lymphatic system are lymphoma, a cancer of B or T lymphocytes, and cancer metastasis, which is responsible for the majority of cancer-related deaths (8).

## Structural differences between vascular and lymphatic endothelium

There are several distinct structural and molecular features that differ among vascular and lymphatic ECs. These structural differences are most striking among the vascular capillaries and the initial lymphatics, which have distinct physiological functions. The function of the vascular capillaries is to facilitate the exchange of nutrients, gases, and waste products, whereas the function of the initial lymphatics is the absorption of excess interstitial fluid and protein (5).

The initial lymphatics are blind-ended vessels that are only capable of unidirectional transport of lymph (5). Lymphatic endothelial cells (LECs) lack mural cells, such as pericytes, with an absent or incomplete basement membrane, and attach themselves to surrounding connective tissue with anchoring filaments composed of the glycoproteins Fibrillin or Emilin1 (9) (Fig. 1*D*). Anchoring filaments are thought to prevent collapse of lymphatic vessels and increase interstitial fluid absorption in edematous tissue by holding the vessel lumen open. Another structural characteristic unique to the initial lymphatics is the presence of flap valves (Fig. 1*D*). These valves are formed by overlapping edges of ECs that open to allow influx of interstitial fluid into the initial lymphatics and prevent it from leaking into the interstitium (6). To further facilitate the unidirectional transport of the fluid, the initial lymphatics drain into collecting lymphatics with intraluminal valves (Fig. 1*D*).

**Figure 2:**
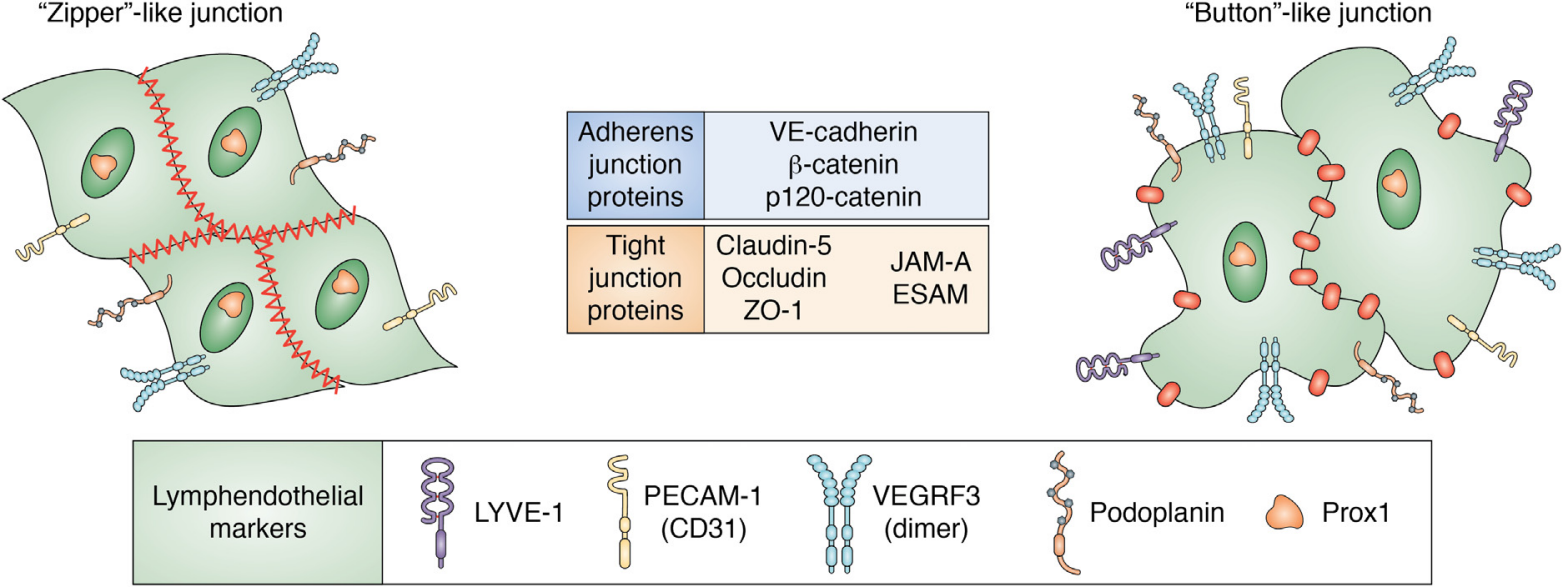
**Schematic representation of endothelial cell–cell junctions and associated proteins in lymphatic endothelial cells.** Lymphatic endothelial cells form specialized junctions to maintain the integrity of blood and lymphatic vessels. Zipper-like junctions found in collecting lymphatics and button-like junctions of the initial lymphatics are formed by the adherens junction proteins VE-cadherin, p120-catenin, and β-catenin, which bind to each other and the actin cytoskeleton. Tight junctions are formed by the transmembrane proteins Claudin-5 and Occludin, and the cytoplasmic protein ZO-1, which regulate paracellular permeability. Additional tight junctional proteins include junctional adhesion molecule (JAM)-A and endothelial cell-selective adhesion molecule (ESAM), which are also involved in leukocyte transmigration. Lymphatic vessels are distinguished by the expression of specific markers, including lymphatic vessel endothelial hyaluronan receptor 1 (LYVE-1), platelet endothelial cell adhesion molecule (PECAM)-1, prospero homeobox protein (Prox) 1, and vascular endothelial growth factor receptor (VEGFR) 3, and podoplanin. Together, these junctional and marker proteins play critical roles in maintaining the structural and functional integrity of blood and lymphatic vessels.

In addition to the unique structural features that distinguish vascular endothelial cells (VECs) and LECs, there are several different molecular markers that can be used to define them (Fig. 2). Platelet endothelial cell adhesion molecule-1 (PECAM-1 or CD31) is expressed on all cells within the vascular compartment (Fig. 2), whereas the lymphatic vessel endothelial hyaluronan receptor 1 (Lyve-1), a transmembrane glycoprotein that binds hyaluronan, is exclusively on the surface of LECs (Fig. 2), sinusoidal ECs in spleen, and syncytiotrophoblast in placenta (10). Another marker of LECs is podoplanin (Fig. 2), a surface glycoprotein that interacts with galectin-8, and together they are able to increase LECs adhesion (11). An additional marker of LECs is Prox1 (Fig. 2), the transcription factor expressed among the population of ECs within an embryo that ultimately develop into the lymphatic system (5). Knockout of Prox1 expression prevents the development of the lymphatic system without affecting that of the vascular system (12). In addition, knockdown of Prox1 expression in adult LECs led to the dedifferentiation of LECs to VECs, indicating that continuous expression of Prox1 expression is required to maintain lymphatic identity (13). Finally, VEGR-3 is another protein typically associated with the lymphatic system (Fig. 2). VEGFR-3 is a receptor tyrosine kinase that is activated by the binding of its ligands, VEGF-C and VEGF-D, which drive lymphangiogenesis (5). VEGFR-3 has been used as a marker for LECs, although it does appear to be expressed on a subset of ECs, including those involved in angiogenesis (14). Single-cell RNA sequencing of gene expression identified new markers and functions of LECs and provided insights into the diversity between VECs and LECs (15, 16). Although much has already been revealed about heterogeneity of VECs and LECs, further studies are needed to examine the contributions of organ-specific microenvironments and additional features of the lymphatics.

## Endothelial junctions

The regulation of vascular and lymphatic endothelial permeability and barrier stability is in large part mediated through two different types of cell–cell junctions between adjacent cells: tight junctions and adherens junctions. The junctional complexes involved in cell–cell adhesion are made up of transmembrane proteins with extracellular domains that can homodimerize with other molecules of the same type located on the surface of adjacent cells. The cytoplasmic domains of these proteins typically bind to a variety of adapter molecules that connect them to the actin cytoskeleton. In ECs, the tight junctions and adherens junctions are typically dispersed throughout the cell–cell contacts (3).

Tight junctions are responsible for maintaining selective permeability of the endothelial barrier, allowing passage of positively charged ions and small molecules through the endothelial barrier *via* paracellular transport (3). Because different tissues in the body require different degrees of vascular permeability to maintain homeostasis, expression and distribution of tight junction proteins is tissue specific. The protein components of tight junctions include claudins, occludins, and junctional adhesion molecules (JAMs) (17) and the endothelial cell-selective adhesion molecule (Fig. 2). Of these, claudins are the most common tight junction protein found in ECs (17) and claudin-5 particularly plays an important role in restricting diffusion of large molecules through paracellular space (3). Claudins are transmembrane proteins with four extracellular domains, one of which mediates dimerization with claudin molecules on the surface of adjacent cells (Fig. 2). The cytoplasmic tail of claudins has a PDZ domain, which allows them to associate with ZO-1, a protein that anchors claudins to the actin cytoskeleton (3). Occludins are involved in the maintenance of barrier integrity and are structurally quite like claudins. They also have four extracellular domains and dimerize with other occludin molecules on adjacent cells (17). Their cytoplasmic C-terminal domains also interact with ZO-1 to facilitate binding of occludin to the cytoskeleton (3). The JAMs are single-span transmembrane proteins that interact with JAMs on adjacent cells to stabilize tight junctions (17). Their cytoplasmic tails have a PDZ binding motif, which assists with the binding of various proteins, including ZO-1. There are three different types of JAMs, JAM-A, JAM-B, and JAM-C. Of these, JAM-A and -C are expressed on both epithelial and endothelial cells, whereas JAM-B is found only on ECs (3). In addition, JAM-C is more highly expressed on LECs than VECs. The heterogeneous expression of JAM proteins across cell types and locations may be responsible for differences in endothelial barrier integrity between VECs and LECs.

Adherens junctions are complexes that are composed primarily of VE-cadherin on ECs. Cadherins are transmembrane proteins with five extracellular cadherin-like repeats, through which they are able to homodimerize with other cadherin molecules on the surface of adjacent cells (18). The cytoplasmic tail of VE-cadherin contains two domains: a juxtamembrane domain that binds to β-catenin and a C-terminal domain that binds p120-catenin and plakoglobin (18). Both β-catenin and plakoglobin are able to bind to α-catenin, which connects the complex to the cytoskeleton (18). ECs but not LECs also express N-cadherin, which is typically distributed throughout the cell membrane rather than being localized within adherens junctions complexes.

A structural difference between the cell–cell junctions of VECs and LECs is their distribution across the cell surface (19). Initial lymphatics display specialized junctions, called “button-like junctions” (Fig. 1*D*), comprising VE-cadherin and tight junction proteins (claudin-5, occludin, ZO-1, and JAM-A) that attach adjacent cells at the base of interdigitating flaps between cells (20). In intestinal villi, openings between button junctions in intestinal lymphatic vessels called lacteals also serve as entry routes for chylomicrons. The endothelium of collecting lymphatics does not possess these types of junctions and instead displays continuous zipper-like endothelial junctions (Fig. 1*D*) like those of VECs to transport lymph to circulation without leakage. It has been suggested that zipper-like junctions of collecting ducts are the default type of junction produced by LECs, and it takes time for initial lymphatics to develop more specialized button-like junctions (20).

Recent elegant studies provide compelling evidence that the small, monomeric GTP RhoA and its effector Rho- associated protein kinase (ROCK) regulate LECs junction integrity in different vascular beds (21, 22, 23). Inhibition of RhoA/ROCK signaling or activation of VEGFR2/3 signaling induces button to zipper conversion in lacteals and regulates chylomicron uptake suggesting that lacteal junction zippering can protect from diet-induced obesity (21, 24). Furthermore, EphrinB2/EphB4 signaling regulates claudin-5 at LECs junctions through the Rho/ROCK pathway and is necessary for stabilization of cell junctions in collecting lymphatics (23). Another study further identified the transcription factors FOXC1 and FOXC2 as key mediators of mechanotransduction in the control of cytoskeletal organization and RhoA/ROCK signaling in valve LECs (22). Further advances are needed to better explain the mechanisms by which signaling pathways control LECs junction integrity and for a comprehensive understanding of button junction formation, maintenance, and plasticity.

## S1P metabolism and signaling

**Figure 3:**
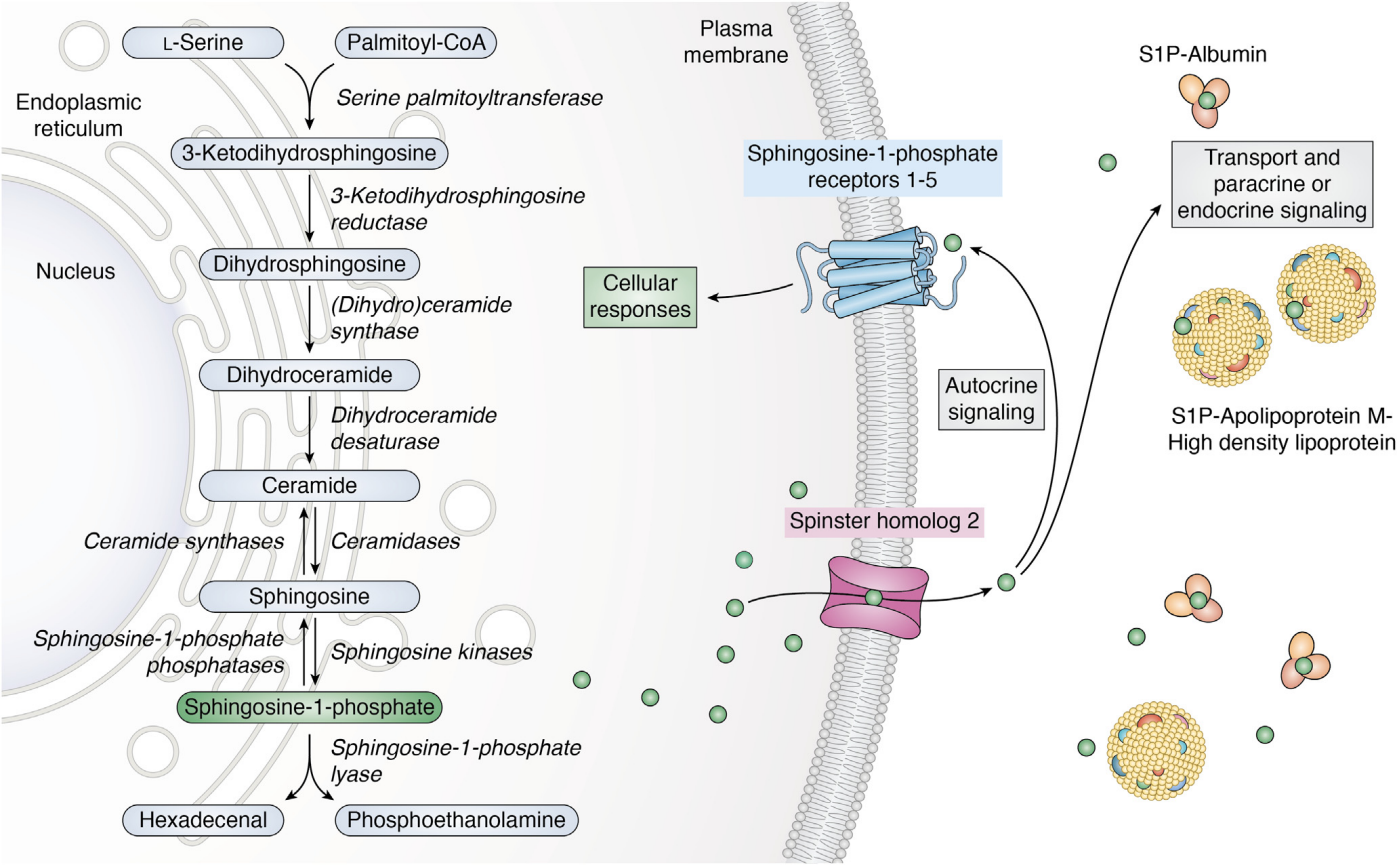
**Simplified overview of bioactive sphingolipid metabolism and transport and inside-out signaling of S1P.** Biosynthesis of sphingolipids is initiated at the endoplasmic reticulum with the condensation of L-serine and palmitoyl-CoA catalyzed by the enzyme serine palmitoyltransferase to form 3-ketodihydrosphingosine. The next step involves the reduction of 3-ketodihydrosphingosine to dihydrosphingosine by 3-ketodihydrosphingosine reductase. Dihydrosphingosine is then converted into dihydroceramide by ceramide synthases. Dihydroceramide is further modified by dihydroceramide desaturase to produce ceramide. Ceramide serves as a precursor for other sphingolipids such as sphingomyelin, glucosylceramide, and galactosylceramide (not shown). Sphingosine is produced by the breakdown of ceramide by ceramidases. Sphingosine is further phosphorylated by sphingosine kinases to produce sphingosine-1-phosphate (S1P). Levels of S1P are regulated by sphingosine-1-phosphate phosphatases and sphingosine-1-phosphate lyase. S1P can be degraded to hexadecenal and phosphoethanolamine. S1P can be transported outside of cells *via* spinster homolog 2 and binds to its specific receptors, sphingosine-1-phosphate receptor 1 to 5, to elicit cellular responses. S1P can act as an autocrine and paracrine signaling molecule in the surrounding, and it can also act at distant locations. In the bloodstream, most S1P is bound and carried by apolipoprotein M in high-density lipoproteins or by albumin. Regulation of sphingolipid metabolism is crucial for maintaining cellular homeostasis and preventing disease development.

Sphingosine-1-phosphate (S1P) is a bioactive sphingolipid metabolite that has been implicated in regulation of numerous important biological processes essential for lymphocyte trafficking, vascular maturation, homeostasis, and diseases (25, 26). S1P is produced by phosphorylation of sphingosine catalyzed by two sphingosine kinases (SphK1, SphK2) (25). At the endoplasmic reticulum, S1P can be dephosphorylated by specific phosphatases or irreversibly degraded by S1P lyase into phosphoethanolamine and hexadecenal, which can be used as substrates for the production of glycerophospholipids (27) (Fig. 3).

Various stimuli activate SphK1 inducing its translocation to the plasma membrane where its substrate sphingosine resides leading to increased formation of S1P. S1P is exported from cells, and many of the actions of S1P are mediated by its binding to five specific G protein–coupled receptors, designated S1PR1-5 (27) (Fig. 3). This paradigm has been coined inside-out signaling by S1P (27). These GPCRs signal through cytoplasmic bound G proteins consisting of α-, β-, and γ-subunits. The functional and structural classification of G proteins depends on their α-subunit and gives rise to the classification of G_i_, G_s_, G_q_, and G_12/13_. They can act independently, synergistically, or antagonistically. S1PRs are distributed differently across cell types, and coupling to specific G proteins leads to activation of several downstream signaling pathways, accounting for the wide variety of effects mediated by S1P. Previous studies have also identified important intracellular targets for S1P (27).

S1P concentrations in blood and lymph are much greater than in lymphoid tissues, forming an S1P gradient that attracts lymphocytes from the thymus or secondary lymphoid tissues and promotes their S1PR1-dependent egress into the blood or the lymph (27). Owing to its negatively charged phosphate group, S1P is unable to independently cross the plasma membrane and requires a transporter to be exported from the cell. In both VECs and LECs, the primary transporter for S1P is spinster homolog 2 (SPNS2) (28) (Fig. 3). In the lymphatics, LECs are responsible for transport of S1P into lymph (29). In contrast, VECs export some of the S1P found in the blood but the majority is due to S1P released from erythrocytes and platelets *via* Mfsd2b transporter (30, 31).

## S1PRs in regulation of vascular endothelium

**Figure 4:**
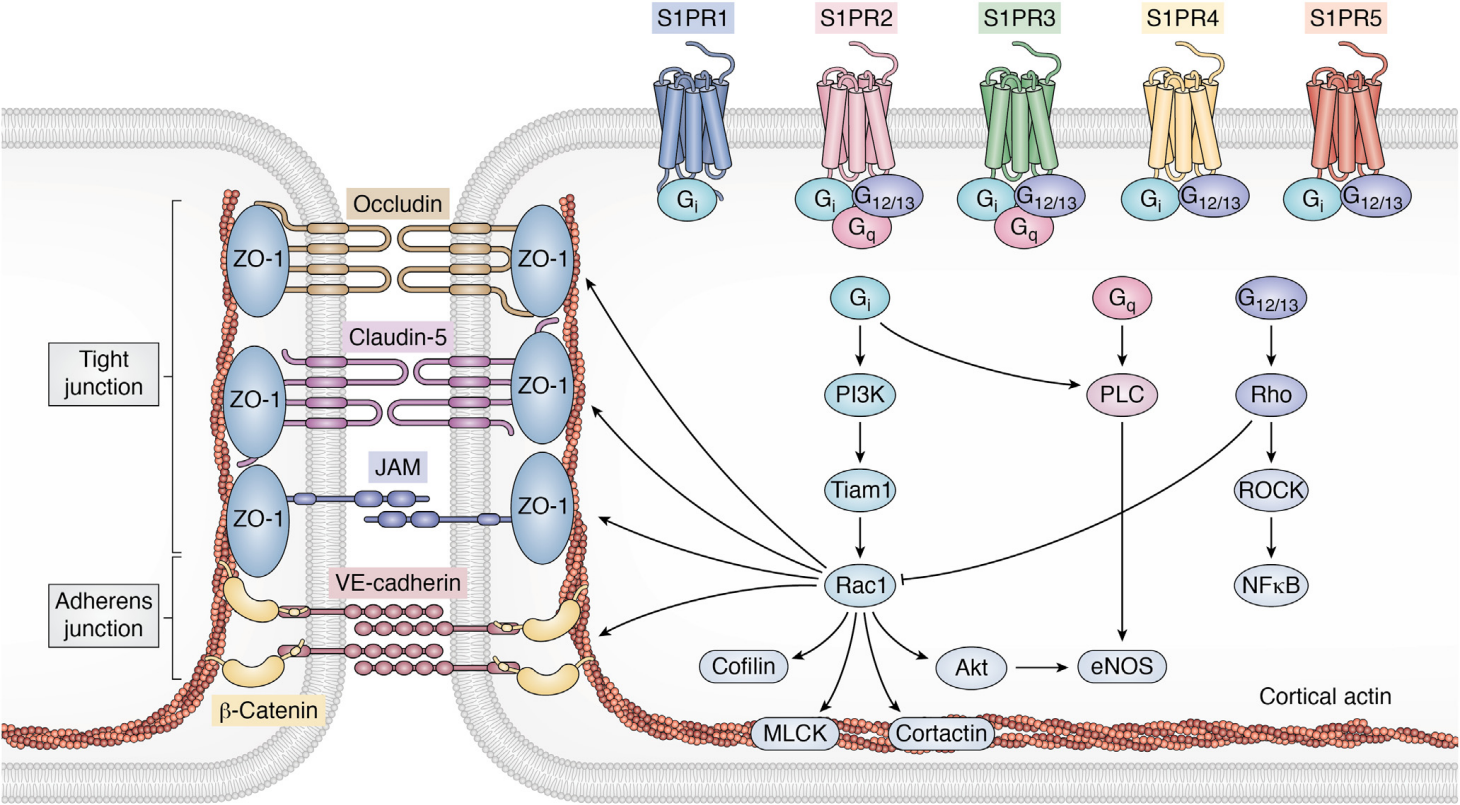
**Sphingolipid signaling regulates tight and adherens junctions through S1PRs.** S1P is a ligand for five different sphingolipid receptors (S1PR), namely, S1PR1, S1PR2, S1PR3, S1PR4, and S1PR5. All S1PRs can signal *via* G_i_, S1PR2 and S1PR3 also *via* G_q_, and S1PR2-4 in addition *via* G_12/13_. G_i_ activation leads to stimulation of phosphoinositide 3-kinases (PI3K) and Tiam1, which stimulate Rac1. Rac1, in turn, interacts with various downstream effectors, including cofilin, myosin light chain kinase (MLCK), cortactin, Akt, and endothelial NO synthase (eNOS), and regulates the actin cytoskeleton, the cortical actin ring, tight junction proteins, including ZO-1, JAM, claudin-5, and occludin, and the adherens junction proteins beta-catenin and VE-cadherin at the cell periphery. G_i_-coupled signaling can also activate phospholipase C (PLC). Likewise, G_q_ activation signals through PLC to activate eNOS forming NO that is important for blood pressure and flow. G_12/13_ activation signals through Rho, which inhibits Rac, leading to activation of Rho- associated protein kinase (ROCK) and nuclear factor “kappa-light-chain-enhancer” of activated B cells (NF-κB). This figure highlights the complexity of sphingolipid signaling in regulation of junctional complexes and underscores the importance of understanding these mechanisms in the context of various physiological and pathological conditions regulated by barrier functions.

VECs are known to express high levels of S1PR1 and relatively low levels of S1PR2 and S1PR3 (32). All three S1P receptors on VECs are coupled to G_i_, but S1PR2 and S1PR3 are additionally coupled to G_q_ and G_12/13_ (Fig. 4). S1P in the blood, which is bound to both albumin and apolipoprotein M in high-density lipoprotein (Fig. 3), activates S1PR1 on VECs to maintain endothelium barrier function by promoting cell–cell interactions (25). There have been numerous studies on the role of S1P in barrier function (reviewed in (26, 33)), although some of the exact molecular mechanisms involved are still being resolved. The ability of S1P to regulate the vascular endothelial barrier was originally described by Proia’s group who found that S1PR1 knockout is lethal due to leaky blood vessels and deficiency in vascular maturation (34). Additional deletion of S1PR2 and S1PR3 in double and triple knockout mice accelerated these phenotypes during embryonic development suggesting a mutual relation of these receptors (35). Indeed, it was confirmed that stimulation of a monolayer of ECs with S1P increased the trans-monolayer electrical resistance. This increase in barrier function was mediated by S1PR1 leading to Rac activation and recruitment of the actin filament regulatory protein, cofilin (36). Subsequently, it was shown that S1P enhanced recruitment of S1PR1 into caveolin-enriched microdomains and promoted activation of PI3 kinase and production of PIP3. This in turn led to recruitment of Tiam1, a GEF that activated the GTP-binding protein Rac1, resulting in the translocation of that activated Rac1 and the cytoskeletal protein α-actinin causing rearrangement of cortical actin to increase ECs barrier function (37). Rac activation is also coupled to myosin light chain kinase and phosphorylation of myosin light chain and cortactin leading to polymerization of cortical actin and reorganization of tight junctions (38) (Fig. 4).

To determine whether S1P enhances barrier function *in vivo*, mice lacking S1P selectively in plasma were produced (39). These mutant mice had increased vascular leak associated with increased interendothelial cell gaps in venules. The increased leak was reversed by transfusion with wildtype erythrocytes that restored plasma S1P or by treatment with a S1PR1 agonist. Two signaling models were proposed to explain the mechanism through which plasma S1P regulates VECs barrier enhancement (39). In the homeostatic model, S1P binds to S1PR1 on the lumenal surface of VECs, providing a constant signal to maintain the endothelial barrier. The dynamic model suggests that S1PR1 is predominantly localized to the basolateral surface of VECs and that leakage of S1P from the plasma through the endothelial barrier causes activation of S1PR1 and thus signals the ECs to form a tighter barrier (39). It was also shown that cell-autonomous production of S1P by ECs enhanced barrier integrity *via* S1PR1, which was strongly dependent on expression of SPNS2 (40). In addition, using S1PR1 signaling reporter mice and selective agonists, it was convincingly argued that brain ECs express S1PR1 on their abluminal surfaces, restricting homeostatic S1P signaling to arteriolar VECs (41). This work also revealed a critical protective role for cerebrovascular autonomous engagement of S1PR1 during cerebral ischemia and that this receptor pool is a potential target for neuroprotection with blood–brain barrier (BBB)-penetrating S1PR1 agonists (41).

S1PR1 signaling in VECs not only stabilizes adherens junctions but also enhances endothelial nitric oxide synthase (eNOS)-derived NO production important for blood flow and pressure (42) and decreases expression of leukocyte adhesion molecules to limit vascular inflammation (43, 44). S1PR1 signaling also suppresses VEGF-induced vascular sprouting (45) by stabilization of junctional VE-cadherin and inhibition of VEGFR2 phosphorylation and downstream signaling (46). Thus, S1PR1 acts as a vascular-intrinsic stabilization mechanism to protect developing blood vessels from aberrant angiogenesis (46). However, S1PR1 on VECs also supports VEGFR2-mediated angiogenic signaling during tumor growth (47). Nevertheless, S1PR1 on ECs is necessary for tumor vascular normalization to allow improved blood circulation and enhance antitumor therapy in mouse models (48).

Hepatocyte growth factor, one of the major growth factors for VECs, stimulates their growth and migration by activation of SphK1 and generation of S1P that is transported out of cells by SPNS2 to activate S1PR1 (49). Recently, it was shown that the transcription factors EGR1 and STAT3 are activated during ECs injury, leading to upregulation of *Sphk1* and *Spns2* expression. The increased S1P in turn amplifies programming of S1PR1^+^ expressing ECs that restores vascular integrity following endotoxemia (50). This might be relevant to sexual dimorphism of cardiovascular disease as estrogen stimulates and increases S1P production in VECs (51). S1PR1 activation also enhances expression of SR-BI (scavenger receptor BI) and promotes binding and transendothelial transport of high-density lipoprotein by human aortic ECs, indicating that high-density lipoprotein, in contrast to albumin, passes endothelial barriers by specific mechanisms rather than passive filtration (52).

In contrast to S1PR1, ligation of S1PR2 by S1P activates RhoA/ROCK signaling to increase endothelial permeability and reduce translocation of VE-Cadherin to adherens junctions and actin polymerization (53, 54, 55) (Fig. 4). In addition, S1PR2 induces endothelial inflammation by activation of stress-activated kinase p38 and NF-κB in mice (56). Similar effects were noted *in vivo*, as deletion of S1PR2 in mice had a protective effect on LPS-induced barrier and BBB disruptions as well as neutrophil infiltration (56, 57, 58). Moreover, inhibition of S1PR2 suppresses endothelial dysfunction and atherogenesis in ApoE KO mice, which accumulate cholesterol ester particles in the blood and promote development of atherosclerotic plaques (59).

Involvement of S1PR3 in endothelial barrier functions is more controversial. S1PR3 can interact with Gq leading to induction of calcium signaling that activates the RhoA/ROCK pathway as well as eNOS (Fig. 4). Reciprocally, NO generation disrupts adherens junctions by nitrosylation of p190RhoGAP, a GTPase that blocks RhoA activity (33). Moreover, it was reported that deletion of S1PR3 in mice increases lesion size in carotid arteries following ligation injury (60); however, it has the opposite effect in denuded iliac-femoral arteries, where it decreases neointimal lesion size (61). It is possible that S1PR3 inhibits lesion growth in carotid arteries because of its function in ECs, whereas S1PR3 is anti-inflammatory by activating eNOS (62). Nevertheless, S1PR3 on ECs promotes leukocyte rolling by inducing mobilization of P-selectin to the cell surface, which is an opposite effect to S1PR1 signaling (63). However, during systemic inflammation, the barrier-stabilizing effect of increased S1P levels by S1P lyase inhibition (64) is abolished in S1PR3 global knockout mice (65). It is therefore possible that the effects of S1PR3 potentially overlap with those of S1PR1 or S1PR2 depending on the cell type, expression of the receptors, and the context. Recently, it was suggested that, in homeostatic conditions, expression of S1PR4 on the abluminal surface of ECs similar to S1PR1, and in contrast to S1PR2, promotes BBB integrity by stimulating Rac and increasing adherens junction and tight junctions (66). However, under pathological conditions and leakage of plasma S1P, increased abluminal S1P decreases expression of S1PR4 compared with S1PR2, causing an increase in BBB-disruption associated with RhoA/ROCK and NF-κB signaling (66). In addition, siponimod (BAF-312), a functional antagonist of S1PR1 and S1PR5, attenuates disruption of BBB induced by inflammatory conditions by interaction with both S1PR1 and S1PR5, increasing expression and function of endothelial tight junctional proteins, ZO-1 and claudin-5 (67) (Fig. 4). This supports the notion that changes in receptor expression during disease conditions could contribute to exacerbation of vascular injury by favoring distinct signaling pathways.

## S1P regulation of lymphatic endothelium

Not much is known about the response of the lymphatic endothelium to S1P and its role in regulation of barrier functions. LECs express high levels of SphK1 and SphK2, as well as Spns2 (29). They also express high levels of S1PR1 and S1PR3, although to a lesser extent (68).

SphK2 null mice with a conditional knockout of lymphatic SphK1 had a loss of S1P in lymph, but not in plasma, causing altered morphology of the initial lymphatic vessels in nonlymphoid tissues with a less organized VE-cadherin distribution at cell–cell junctions. These junctions were also less defined with fewer or more diffuse “buttons” (29). These findings support a role for S1P in lymphatic vessel maturation and imply that LECs within the lymph node and in the vasculature of nonlymphoid tissues may be regulated differently by S1P. This study also demonstrated that LECs are an *in vivo* source of S1P required for lymphocyte egress from lymph nodes and Peyer’s patches, nodules of lymphatic cells that aggregate to form patches (29). *Spns2* knockout mice also have a robust reduction in lymph S1P and decreased circulating lymphocytes (69). Moreover, lymph nodes from *Spns2* knockout mice have aberrant lymphatic sinuses that appeared collapsed, with reduced numbers of lymphocytes (70). Likewise in Lyve1;Spns2^Δ/Δ^ conditional knockout mice, high endothelial venules (HEVs) in the lymph nodes (Fig. 1, *B*), which express Lyve-1 during development, appeared apoptotic and were impaired in function, morphology, and size (71). It was shown that high-endothelial cell-derived S1P was necessary for maintaining HEV integrity through autocrine regulation of S1PR1-Gi signaling and to facilitate interaction of dendritic cells with HEV (71). Moreover, engagement of the transmembrane O-glycoprotein podoplanin expressed on fibroblastic reticular cells that surround HEV with C-type lectin-like receptor 2 (CLEC-2) on extravasated platelets induced their local release of S1P. This promoted VE-cadherin expression on HEV critical for its integrity during immune responses (72). However, others have suggested that stimulation of α9 integrin on LECs by its ligand tenascin-C enhances local S1P secretion without affecting S1P synthesis and/or degradation (73). In contrast, it was proposed that the alpha subunit of the IL-4 receptor (IL4RA) on LECs regulates *Sphk1* expression and S1P release from LECs to promote T-cell egress during graft-versus-host disease (74). However, arthritis inflammation-induced autophagy in LECs promoted degradation of SphK1 and decreased S1P production. Consequently, pathogenic Th17 cell migration toward LECs-derived S1P gradients and egress from lymph nodes, as well as arthritis, was reduced (75). Surprisingly, although the majority of studies clearly showed that LECs provide S1P to lymph (29, 30, 71, 74, 75), it was recently suggested that, during an immune response, hematopoietic cells, particularly inflammatory monocytes, are the source of this S1P (76). S1P secreted from LECs by the Spns2 transporter signals through S1PR1 on T cells, not only to guide their exit from lymph nodes during an immune response (77) but also to promote mitochondrial function and survival of naive T cells (78).

**Figure 5:**
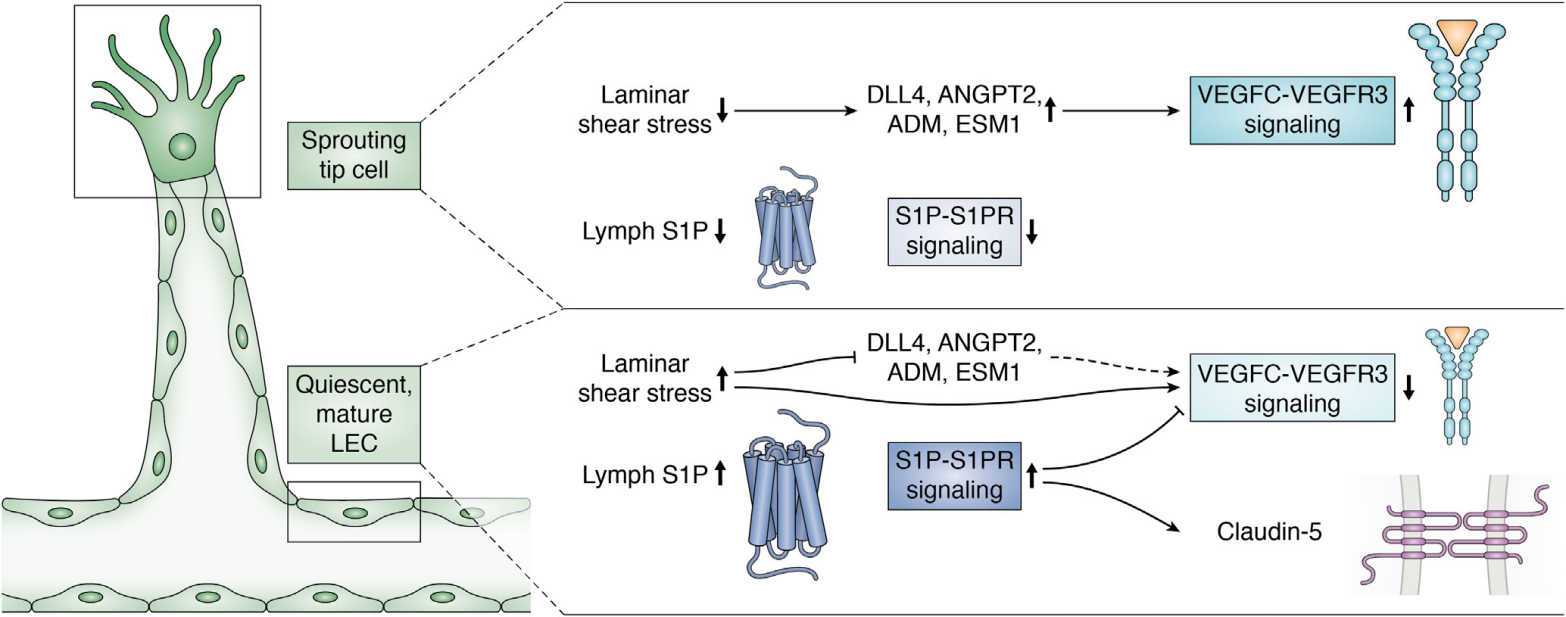
**Regulation of lymphatic vessel sprouting and maturation by laminar shear stress and S1P signaling.** In the absence of laminar shear stress or lymph S1P, the sprouting tip cell expresses the tip cell marker delta-like 4 (DLL4) and angiopoietin-2 (ANGPT2), adrenomedullin (ADM), and endothelial cell specific marker 1 (ESM1), which promote sprouting *via* vascular endothelial growth factor (VEGF) C and vascular endothelial growth factor receptor (VEGFR) 3 signaling. Mature, quiescent LECs are exposed to laminar shear stress and lymph S1P. Laminar shear stress inhibits DLL4, ANGPT2, ADM, and ESM1, while VEGFC-VEGFR3 signaling is activated. S1P-S1PR1 signaling counteracts VEGFC–VEGFR3 signaling to prevent sprouting and regulate the quiescent state of LECs in lumenized lymphatic vessels. In addition, S1PR1 signaling inhibits RhoA activity, enabling claudin-5 recruitment to the plasma membrane to form tight junctions that promote lymphatic vessel maturation.

It has long been suspected that S1PR1 is responsible for S1P-mediated lymphangiogenesis, the growth of lymphatic vessels (68, 79). Moreover, S1PR1 regulates the directional migration of LECs in response to laminar shear stress (80). This is important, as LECs that trail the growing front are exposed to S1P and laminar shear stress and remodeled into stable vessels. In contrast, tip LECs at the growing front that sprouts by forming filopodia are not exposed to lymph S1P or laminar shear stress. The role of S1PR1 in coordination of these opposed responses of lymphatic sprouting and maturation was recently elucidated. It was shown that during lymphangiogenesis, delta-like 4 (DLL4), a tip cell marker, promotes sprouting by enhancing VEGFC–VEGFR3 signaling, perhaps *via* activation of Notch1 (81) (Fig. 5). However, in lumenized LECs, laminar shear stress inhibits expression of tip cell markers including DLL4 but upregulates VEGF-C/VEGFR3 signaling, which is counteracted by S1P-S1PR1 signaling, thereby preventing sprouting from quiescent lymphatic vessels (81) (Fig. 5). In addition, S1PR1 enhances lymphatic vessel maturation by inhibiting RhoA/ROCK signaling to promote formation of claudin-5-positive cell–cell junctions (81) (Fig. 5). These results highlight the distinct roles of S1PR1 in the lymphatics compared with the blood vasculature. Interestingly, binding of lysophosphatidic acid to its receptor LPAR1 in LECs of lymph nodes enhances β-arrestin recruitment to S1PR1 and inhibits S1P-induced Gαi signaling, causing remodeling of junctions from continuous to punctate structures (82). This heterotypic inter-GPCR cross talk suppressed S1PR1 signaling and produced porous junctional architecture of sinus-lining LECs, to enable efficient lymphocyte trafficking (82). Moreover, it was shown that a loss of S1PR1 with high S1PR1/β-arrestin coupling in LECs resulted in transcriptional alterations of genes associated with lymphangiogenesis (*Kdr, Prox1, Lyve1, Nr2f2*) and genes related to immune responses (*Irf8, Lbp, Il7, Il33 Ccl21, Tnfaip8l1)* (83).

Intriguingly, it was suggested that S1PR2, and not S1PR1, on LECs was required for migration of CD4 T cells across LECs and into lymphatic vessels and draining lymph nodes (84). To evaluate the role of S1PR2 in lymphatic endothelium, junctions of LECs were examined after treatment with an S1PR2 inhibitor (84). Inhibition of S1PR2 altered VCAM-1 and increased VE-cadherin expression in buttons, as well as increased Occludin and ZO-1, proteins involved in the formation of tight junctions (84). Thus, S1PR2 signals through ERK to regulate the density and distribution of button and zipper endothelial junctions, lymphatic permeability, and interaction of CD4 T cells and LECs during translymphatic endothelial migration (84).

## Conclusions and future perspectives

The discussed studies demonstrated distinct roles for S1PRs in LECs functions and suggest additional levels of integrated processes in responses to S1P in both LECs and T cells. Future studies are needed to understand how and why multiple receptors are used simultaneously to direct interactions between T cells and LECs and to determine if efferent and afferent lymphatics function similarly, or whether other lymphatics beds have unique responses to S1P.

Abundant new evidence demonstrates that the plasticity of the lymphatics and specialized LECs zipper- or button-like junctions are tightly regulated to maintain lymphatic vessel integrity and function (21, 22, 23). For example, lipid absorption by intestinal lacteals is influenced by button conversion to zippers, explaining how junctional modification in lymphatics of one organ leads to systemic effects (21, 23, 24). Further understanding of the functions of S1PRs in developmental and pathophysiological processes and in regulation of zipper- or button-like junctions, and how expression of S1PRs contributes to conversion of buttons to continuous zipper junctions or reversion back to buttons, is needed.

Additional comprehensive studies using combinations of physiological approaches in knockout mouse models together with single-cell RNA sequencing of gene expression in LECs are needed to more fully understand the mechanism by which S1PR-regulated signaling pathways, in addition to RhoA/ROCK signaling, are involved in regulating functions of lymphatic vessels and lymphatic plasticity. Such studies may have implications for the development of potential therapeutics targeting S1PRs to modulate LECs junctions, permeability, and function in diseases with dysregulated lymphatic barrier function.

## Conflict of interest

The authors declare that they have no conflicts of interest with the contents of this article.
